# TIM-1 promotes infection with mosquito cell-derived alphaviruses through virion-associated phospholipids

**DOI:** 10.1080/22221751.2026.2673648

**Published:** 2026-05-13

**Authors:** Ju Eun Yoo, Anja C. M. de Bruin, Hanna Reßin, Mindaugas Paužuolis, Lifeng Liu, Anja Moosmann, Miriam Becker, Lisa Lasswitz, Rüdiger Groß, Gisa Gerold

**Affiliations:** a Department of Biochemistry, University of Veterinary Medicine Hannover, Hannover, Germany; b Research Centre for Emerging Infections and Zoonoses, University of Veterinary Medicine Hannover, Hannover, Germany; c Institute of Molecular Virology, Ulm University Medical Center, Ulm, Germany; d Department of Clinical Microbiology, Umeå University, Umeå, Sweden; e Wallenberg Centre for Molecular Medicine (WCMM), Umeå University, Umeå, Sweden; f Institute of Virology, Department of Hygiene, Microbiology and Virology, Medical University of Innsbruck, Innsbruck, Austria

**Keywords:** Alphavirus, mosquito, TIM-1, ONNV, phosphatidylethanolamine, PS receptor

## Abstract

Human T-cell immunoglobulin and mucin domain 1 (TIM-1) facilitates infection of re-emerging viruses, including alphaviruses, through its phosphatidylserine (PS) binding domain. Although alphaviruses are transmitted to humans via mosquito bite, it is unclear whether mosquito-derived viruses also use TIM-1 to infect human cells. Since viruses acquire their PS-containing envelope from the host cell and insect cell membranes differ in lipid composition from mammalian membranes, we here investigate the role of TIM-1 at the mosquito-mammalian interface. We show that TIM-1 promotes infection with mosquito cell-derived, replication-competent alphaviruses, including Chikungunya virus, O’nyong’nyong virus (ONNV), and Sindbis virus. The TIM-1 PS-binding domain is essential for enhancing mosquito cell-derived ONNV infection as shown by TIM-1 mutagenesis. According to untargeted lipidomics, mosquito cell-derived ONNV virions contain higher levels of phosphatidylethanolamine (PE) and PS compared to mammalian cell-derived ONNV. Notably, TIM-1 engages PE as well as PS, as demonstrated by liposome competition. PS decarboxylase experiments suggest that PS is an important mediator of mosquito-cell derived ONNV entry into mammalian cells. Taken together, our data show that TIM-1 promotes mosquito cell-derived alphavirus infection of mammalian cells. This work provides implications for the understanding of the TIM-1 ligand repertoire and advances our understanding of arbovirus transmission at the mosquito-mammalian interface.

## Introduction

Alphaviruses (family *Togaviridae*, genus *Alphavirus*) are small positive-sense, single-stranded RNA viruses [1]. They are primarily transmitted by haematophagous mosquitoes and infect a broad range of vertebrate hosts. The latter include nonhuman primates, horses, birds, rodents, marsupials, and humans. Alphaviruses are categorized into Old World and New World viruses based on the geographical location where they were first identified. In humans, Old World alphaviruses are mainly associated with arthritogenic symptoms and New World alphaviruses with encephalitic symptoms [2]. Since the mid-2000s, Chikungunya virus (CHIKV) has received most attention due to its global re-emergence [3].

Alphaviruses enter cells primarily through clathrin-mediated endocytosis [4]. So far, several alphavirus attachment factors and proteinaceous entry receptors have been identified [5–8]. One such factor is the attachment factor T-cell immunoglobulin and mucin domain 1 (TIM-1) [9]. TIM-1 is a type I transmembrane glycoprotein and a member of the TIM protein family, consisting of TIM-1, TIM-3, and TIM-4 in humans. TIMs have an extracellular N-terminus composed of an immunoglobulin variable domain (IgV) and a mucin domain. A single transmembrane domain and a cytoplasmic domain compose the C-terminus [10]. TIM proteins are primarily expressed in immune cells [10] and in mucosal epithelial cells [11,12]. All human TIMs bind phosphatidylserine (PS) at their IgV domain and thereby mediate clearance of PS-exposing apoptotic cells [13,14]. Santiago et al. solved the crystal structures of murine TIM proteins and human TIM-4 bound to PS, identifying the metal-ion-ligand binding site (MILIBS) as the site of PS binding [15,16]. Mutations of MILIBS residues disabled PS binding and consequently phagocytosis [14]. The MILIBS also mediates TIM-1’s role as an attachment factor for CHIKV entry in mammalian cells [9,17–19]. These studies support that virion-associated PS is required for virus attachment to TIM-1. Although PS is the conventionally regarded ligand of MILIBS, phosphatidylethanolamine (PE) has also been reported to be a ligand for this site [20].

Alphaviruses bud at the plasma membrane of infected cells. Consequently, the host plasma membrane is integrated into the new particle, forming the viral envelope. As alphaviruses replicate in both mosquito and vertebrate hosts, the source of their viral envelope alternates between a mammalian lipid membrane and a mosquito lipid membrane. Mammalian membranes are enriched in sphingolipids and sterols [21], and the most abundant phospholipids are PE and phosphatidylcholine (PC) [22]. Moreover, the plasma membrane of healthy mammalian cells is asymmetrical at equilibrium state, with PC located on the outer leaflet and PE and PS on the inner leaflet [23]. In insect cells, the plasma membrane lipid composition is notably different [24]. Insect cells are more abundant in PE and PS, and a constitutively active phospholipid scramblase exposes these phospholipids on the outer leaflet of the plasma membrane [25]. Despite these differences, it remained elusive whether alphaviruses derived from mosquito cells bear a TIM-1 ligand in their envelope and interact with TIM-1. In this study, we investigated TIM-1 dependency of mosquito cell-derived alphaviruses. Furthermore, lipid composition of mosquito cell-derived O’nyong’nyong virus (ONNV) particles was compared to that of mammalian cell-derived particles. Subsequently, we analysed which lipids in mosquito cell-derived virions are critical for mammalian cell attachment and entry.

## Materials and methods

### Cell culture and generation of stable cell lines

Baby Hamster Kidney cells (BHK-21, ATCC CCL-10), parental Human Embryonic Kidney cells (HEK 293 T, ATCC CRL-3216; hereafter, abbreviated HEK), HEK cells stably expressing TIM-1 or ΔMILIBS TIM-1 [9] or Mxra8, human dermal fibroblasts (kindly provided by D. Bogunovic) [26], and A549ΔMAVS [27] were maintained at 37 °C with 5% CO_2_, in high glucose Dulbecco’s Modified Essential Medium (DMEM; Gibco) supplemented with 10% fetal calf serum (FCS; Capricorn Scientific), 0.1 mM nonessential amino acids (NEAA; Gibco), 100 U/mL penicillin, and 100 μg/mL streptomycin (Gibco). HEK TIM-1 and HEK ΔMILIBS TIM-1 were maintained in 5 μg/mL blasticidin (Sigma-Aldrich), HEK Mxra8 in 5 μg/mL puromycin (Sigma-Aldrich), HEK TIM-1 Mxra8 in blasticidin and puromycin, and A549ΔMAVS in 2 μg/mL puromycin.

Aag2-AF5 (ECACC 19022601) and C6/36 (ATCC CRL-1660) cell lines were kindly provided by K. Maringer. Aag2-AF5 cells were maintained in GlutaMAX-supplemented Leibovitz’s L-15 Medium (Gibco) supplemented with 10% FCS, 10% tryptose phosphate broth (Gibco), 0.1 mM NEAA, 100 U/mL penicillin, and 100 μg/mL streptomycin. C6/36 cells were maintained in Schneider’s Drosophila Medium (PAN Biotech) supplemented with 10% FCS, 2 mM L-glutamine (Gibco), 0.1 mM NEAA, 1% sodium pyruvate (Gibco), 100 U/mL penicillin, and 100 μg/mL streptomycin. Mosquito cells were maintained at 28 °C.

The gene encoding human Mxra8 (isoform 1 (NM_001282585); codon-optimized; with C-terminal MycFlag tag) was synthesized (Integrated DNA Technologies), cloned into pWPI_IRES_Puro_Ak (from Sonja Best (Addgene #154984)) lentiviral vector, and used to generate stable cells [9].

### Virus production and titration

BHK-21 cells were electroporated with infectious clones **(**Figure S1**)** to yield reporter-tagged, recombinant alphaviruses. Infectious clones of CHIKV-LR2006 OPY1 5’GFP (provided by Graham Simmons) [28], ONNV-2SG-zsGreen (provided by Andres Merits) [29], SINV Toto1101/Luc (provided by Charles M. Rice) [30], VEEV TC-83/GFP (provided by Ilya Frolov) [31], were *in vitro* transcribed with SP6 polymerase and 5’ capped [32]. Twenty micrograms of mRNA were electroporated into 1 × 10^7^ BHK-21 cells in OptiMEM [9]. For pCMV-SFV(Xho-EGFP)4 [33] plasmid DNA was electroporated directly in BHK-21 cells. P0 virus stock was harvested at 48 h post electroporation. To produce P1 virus stocks, confluent cultures of Aag2-AF5 or C6/36 cells were inoculated at MOI:0.1 (based on BHK-21 titre). Alternatively, dermal fibroblasts were inoculated at MOI:1. At 4 h post infection (hpi), the medium was replaced, further incubated until 48 hpi, centrifuged at 400 × g for 7 min, and clarified through 0.45 μm pore size membrane filters. Virus stocks were titrated on BHK-21 cells by endpoint titration to determine the 50% tissue culture infectious dose (TCID_50_) [34] based on fluorescent reporter expression or firefly luciferase activity [9]. TCID_50_/mL titre was computed using the Spearman-Kärber Method [35]. HAdV-C5 (strain adenoid 75) was produced in A549 cells stably expressing parainfluenza virus 5 V protein [36] and TCID_50_ was determined on HuTu80 cells.

### Infection assays

HEK cells were plated overnight at 2 × 10^4^ cells per well on 0.01% poly-ʟ-lysine (Sigma) coated 96-well plates. Subsequently, cells were infected with 10-fold serial dilutions of the indicated virus stocks. At 24 hpi, cells were washed once in PBS, trypsinized, and fixed with 2% PFA. Technical triplicates were pooled in 1% FCS in PBS for flow cytometric analysis on the Attune Nxt or BD FACS Canto II. Live singlets were gated on FlowJo software v10.10.0. The percentage of infected cells was measured per dilution by reporter virus-positive cell population based on mock-infected cells. For dermal fibroblast-derived ONNV, HEK cells were inoculated with 1:5 diluted virus stock and zsGreen expression was monitored on an Incucyte S3 (Sartorius) every 4 h up to 72 hpi. Expression levels were analysed on the platform’s basic analyser software.

For ΔMILIBS experiments, HEK cells were plated overnight at 8 × 10^5^ cells per well on coated 6-well plates, infected with ONNV, and, at 24 hpi, prepared for flow cytometry as described above.

In all infection assays, MOI was calculated according to the BHK-21-based TCID_50_/mL titre.

### Flow cytometric detection of TIM-1 and Mxra8

Cells were detached in cold 0.02% EDTA in PBS and stained with 1 µg/mL rabbit-anti-TIM-1 (ab228973; Abcam), 0.2 µg/mL mouse-anti-Mxra8 (MB-W040-3; MBL Life Science) or isotype controls rabbit IgG (I5006; Sigma) or mouse IgG2α (14-4724-82; Thermofisher) in PBS with 1% FCS for 1 h on ice. Cells were washed twice with 1% FCS in PBS. Goat-anti-rabbit AF488 (A11034; Invitrogen) or goat-anti-mouse-APC (A865; Invitrogen) secondary antibodies were added at 4 µg/mL for 30 min at RT. Cells were washed and fixed with 1% PFA for 20 min on ice. Protein surface expression was determined in the single cell gate using the BD FACS Canto II and analysed using FlowJo software.

### Lipidomics

Purified BHK-21-derived and C6/36-derived ONNV were analysed by untargeted shotgun lipidomics by Lipotype GmbH (Dresden, Germany), as detailed in the Supplementary Methods.

### Liposome formulation

**Figure 4.**
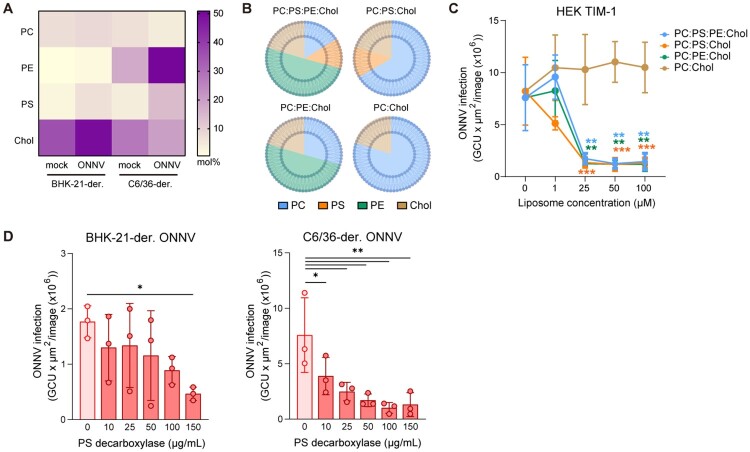
The envelope of C6/36-derived ONNV contains high levels of PE and intermediate levels of PS, which both mediate TIM-1-dependent uptake. (A) Heatmap of mole percentages of PC, PE, PS, and Chol in mock treated supernatants (mock) and virus particles (ONNV) derived from BHK-21 or C6/36. Data represent the median of three technical replicates. (B) Liposome formulations mimicking the PC, PS, PE, Chol mole percentages of C6/36-derived ONNV, and those excluding PS, PE, or both phospholipids. (C) C6/36-derived ONNV infection levels at 12 hpi (MOI:0.5) in HEK TIM-1 incubated in increasing concentrations of the liposomes represented in (B), measured by live cell imaging. Data are mean ± SD of three biological replicates. One-way ANOVA with Dunnett’s compared with untreated control. (D) ONNV stocks were pretreated for 1 h with PS decarboxylase prior to inoculation of HEK TIM-1 at MOI:0.05 (BHK-21-der.) or MOI:0.1 (C6/36-der.) and assessment by live cell imaging. Data are mean infection levels ± SD at 36 hpi. One-way ANOVA with Dunnett’s compared with untreated control.

Lipid components were purchased from Avanti Polar Lipids, Inc. in lyophilized form and then dissolved in chloroform. The indicated mole percent of each lipid component **(**Figure 4(B)**)** was calculated and combined in glass vials, and the organic solvent evaporated under a nitrogen stream for 15-30 min until thoroughly dried. The dried lipid components were resuspended in PBS under shaking for 30 min, resulting in a final molar concentration of 5 mM total lipid. Subsequently, each liposome formulation was extruded 20× through a 0.2 μm membrane using the Avanti Mini Extruder. Formulations were aliquoted and stored at −80 °C until use.

### Liposome competition assays

HEK cells were plated overnight at 2 × 10^4^ cells per well on coated 96-well plates or at 3 × 10^4^ A549ΔMAVS on uncoated plates. The medium was exchanged with 100 µL 2X solution of each liposome formulation diluted in DMEM to yield final liposome concentrations of 1, 25, 50 and 500 µM. Next, 100 µL 2X suspension of BHK-21- or C6/36-derived ONNV in DMEM was added to HEK cells at a final MOI:1 or MOI:0.5, respectively, or at MOI:4 to A549ΔMAVS. At 4 hpi, A549ΔMAVS cells were washed twice and fresh medium was added. ONNV-infected cells were assessed by measuring zsGreen expression by Incucyte S3 up to 72 hpi.

### PS decarboxylase (PSD) treatment

ONNV was treated with a modified, water-soluble and His-tagged version of *Plasmodium knowlesi* PSD (His6-Δ34PkPSD). The enzyme was purified as previously described [37]. HEK TIM-1 cells were plated at 3 × 10^4^ cells per well on coated 96-well plates. The next day, ONNV was incubated with indicated concentrations of PSD in 40 µL PBS for 30 min at 30 °C and 450 rpm on an orbital shaker. To reduce PSD-mediated toxicity, the mixture was diluted 1:40 with DMEM and cells were inoculated at a final MOI:0.05 (BHK-derived ONNV) or MOI:0.1 (C6/36-derived ONNV). At 1 hpi, the inoculum was replaced with fresh DMEM and zsGreen expression assessed by Incucyte S3 up to 48 hpi.

### Duramycin blocking assay

HEK cells were plated at 3 × 10^4^ cells per well on coated 96-well plates. The next day, the virus was incubated with indicated concentrations of Duramycin-LC-Biotin (Molecular Targeting Technologies) in DMEM for 1 h at RT on a tube rotator. Subsequently, cells were inoculated with ONNV at MOI:0.05 or with AdV5 at MOI:1. At 1 hpi, the inoculum was replaced by DMEM and zsGreen expression in ONNV-infected cells was assessed by Incucyte S3 up to 48 hpi. Alternatively, at 24 hpi, AdV5-infected cells were fixed in 5% formaldehyde, permeabilized with 0.5% TritonX-100 for 15 min, blocked with 1% BSA in PBS for 1 h, incubated for 1 h at RT with 0.7 µg/mL mouse-anti-hexon primary antibody (clone 20/11; Sigma; MAB8052) diluted in blocking buffer, washed twice with PBS/0.05% Tween20, followed by 45 min incubation with 1.3 µg/mL goat-anti-mouse-AF488 (A1102; Invitrogen). Hexon staining was assessed by Incucyte S3.

Liposome leakage was assessed using Sulforhodamine B-containing liposomes, prepared as in ***Liposome formulation***, except that the dried lipid components were resuspended in 1 mL of 50 mM Sulforhodamine B. Non-encapsulated Sulforhodamine B was eliminated by passing suspensions twice through PD midi trap Sephadex G-25 columns (GE Healthcare). Particle concentration was measured by nanoparticle tracking analysis. In a 96-well format, 10 µL of duramycin was added to 90 µL of liposomes in PBS (3.3 × 10^11^ particles/mL). Fluorescence was measured in 5 min increments with a microplate fluorescence reader (Cytation 3). To determine 100% liposome leakage, a final concentration of 1% TritonX-100 was added and fluorescence measured for 1 min.

### siRNA treatment of A549ΔMAVS cells

TIM-1 mRNA expression was silenced in A549ΔMAVS cells by reverse transfection in 96-well format using lipofectamine RNAiMAX with Silencer Select siRNAs (s230290, s230291, and s25632) from Thermo Scientific. Pooled siRNA targets (1.5 pmol) and 0.2 μL lipofectamine RNAiMAX were diluted in OptiMEM, mixed, and incubated for 20 min at RT. Non-targeting control siNEG#1 (4390843) was used as negative control. Next, siRNA complexes were added to wells, followed by 3 × 10^4^ A549ΔMAVS cells in DMEM without antibiotics. Cells were incubated for 48 h and inoculated with ONNV at MOI:4 or assessed for TIM-1 surface expression by flow cytometry. ONNV zsGreen expression was assessed by Incucyte S3 up to 72 hpi.

## Statistics

Statistical analyses were performed on GraphPad Prism v10.5.0 with *p* ≤ 0.05 considered significant (*: *p* ≤ 0.05, **: *p* ≤ 0.01, ***: *p* ≤ 0.001, ****: *p* ≤ 0.0001). Area under the curve (AUC) values were calculated per replicate from titration curves on GraphPad Prism v10.5.0, setting the baseline at Y = 0. Graphical representations were generated using GraphPad Prism v10.5.0., BioRender and Adobe Illustrator v25.0.1.

## Results

### Infection of a subset of mosquito cell-derived alphaviruses is facilitated by TIM-1

**Figure 1.**
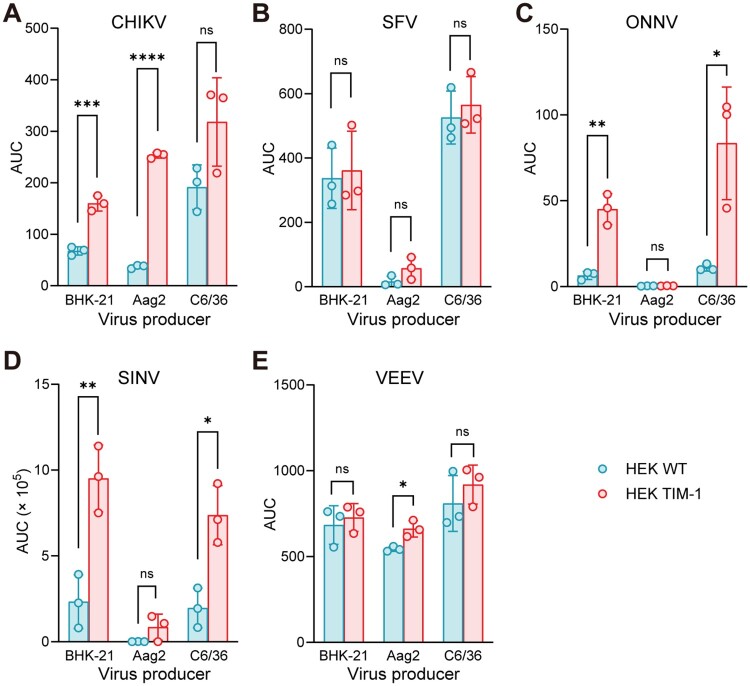
TIM-1 enhances infection by selected mosquito cell-derived alphaviruses in HEK cells. (A–E) Reporter-expressing alphaviruses were produced from BHK-21, Aag2-AF5, and C6/36 cells, titrated on HEK WT and HEK TIM-1, followed by flow cytometry (A–C, E) or luciferase activity (D) analysis at 24 hpi. AUC of titration curves are shown (raw data are shown in Figure S2). Data are mean ± SD of three biological replicates. Unpaired, two-tailed *t*-test. ns: not significant. Aag2: Aag2-AF5.

To evaluate TIM-1 dependency of mosquito-cell derived alphaviruses, we passaged P0 reporter virus stocks of CHIKV, Semliki Forest virus (SFV), ONNV, Sindbis virus (SINV), and Venezuelan equine encephalitis virus (VEEV), all generated in BHK-21 mammalian cells, in Aag2-AF5 and C6/36 mosquito cells. Next, we titrated these mammalian cell- and mosquito cell-derived viruses on TIM-1-negative HEK WT and HEK TIM-1 overexpressing cells [9]. To facilitate comparison of infection levels between HEK WT and HEK TIM-1, we calculated the AUC **(**Figure 1**)** from flow cytometry and luciferase assay titration curves (Figure S2). We observed that three of the tested alphaviruses (CHIKV, ONNV, SINV) showed an increased infectivity on HEK TIM-1 compared to HEK WT, and that this increase was generally independent of producer cell type **(**Figure 1**)**. For each of these three viruses, however, only one of two mosquito-cell-derived stocks reached statistical significance, namely Aag2-derived CHIKV as well as C6/36-derived ONNV and SINV. A TIM-1 mediated increase in infectivity was neither observed for SFV nor for VEEV stocks, except for Aag2-derived VEEV. As we used a hamster cell line to create mammalian-derived virus stocks, we confirmed that ONNV produced in human cells is also TIM-1-dependent by passaging ONNV on human dermal fibroblasts followed by TIM-1 dependency assessment (Figure S3).

In sum, we show that TIM-1 promotes infection of human cells with selected mammalian cell-derived and mosquito cell-derived alphaviruses.

### TIM-1 dependent infectivity is reduced in presence of Mxra8

**Figure 2.**
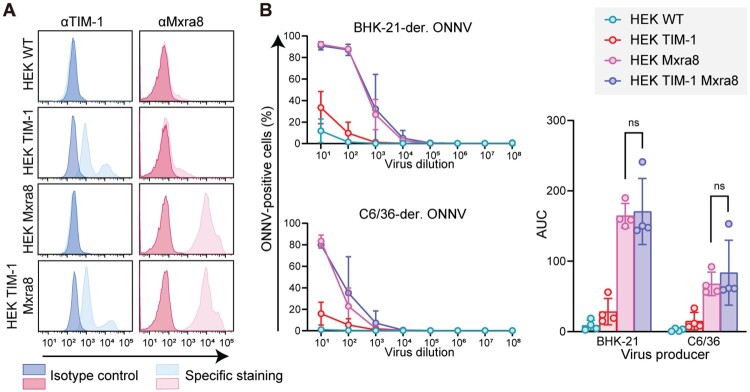
TIM-1 dependency is reduced in the presence of Mxra8. (A) Surface expression of TIM-1 and Mxra8 on transduced HEK cells stably expressing TIM-1, Mxra8, or both. (B) Titration curves of BHK-21-derived (BHK-21-der.) and C6/36-derived (C6/36-der.) ONNV on cells from (A), left, and AUC quantification, right. ONNV-positive cells were determined at 24 hpi by flow cytometry. Data are mean ± SD of four biological replicates. Unpaired, two tailed *t*-test. ns: not significant.

To identify parameters important for TIM-1 dependence, we assessed whether *bona fide* receptor expression modulates TIM-1 reliance. HEK WT cells do not express Mxra8, the receptor for CHIKV, ONNV, and other arthritogenic alphaviruses [6] (Figure 2(A)). We hence generated HEK cells expressing TIM-1, Mxra8 or both (Figure 2(A)) and infected the cells with a serial dilution of ONNV. As expected, Mxra8 presence confers high susceptibility (Figure 2(B)). Interestingly, TIM-1 expression did not enhance ONNV infection in presence of Mxra8 (Figure 2(B)). This suggests that abundant expression of the *bona fide* receptor reduces reliance on TIM-1 during viral entry.

### The MILIBS is essential for TIM-1-dependent ONNV infectivity

**Figure 3.**
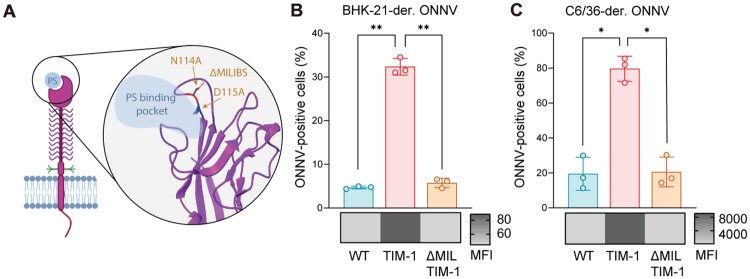
ONNV dependency on TIM-1 is abrogated in HEK ΔMILIBS TIM-1 cells. (A) Schematic representation of TIM-1 and the PS binding pocket with indicated substitutions present in the ΔMILIBS TIM-1 mutant (PDB: 5DZO). (B) HEK WT, HEK TIM-1, and HEK ΔMILIBS TIM-1 cells were infected with (B) BHK-21-derived ONNV (MOI:1) or (C) C6/36-derived ONNV (MOI:0.5) for 24 h and ONNV-positive population was determined by flow cytometry. Data are mean ± SD of three biological replicates. Median fluorescent intensities (MFI) are represented as greyscale heatmap. One-way ANOVA with Tukey’s.

This study is the first to report TIM-1-dependent enhancement of ONNV infection. To test if the MILIBS mediated this enhancement, we used HEK ΔMILIBS TIM-1 cells [9], which display two point mutations (N114A, D115A) in the MILIBS that abrogate PS binding (Figure 3(A)). We did not observe enhanced infection by either BHK-21- or C6/36-derived ONNV in HEK ΔMILIBS TIM-1 cells **(**Figure 3(B and C)**)**. This result confirms the importance of the MILIBS in TIM-1-dependent infectivity and indicates that a virion-associated phospholipid is necessary for C6/36-derived ONNV entry via TIM-1.

### Mosquito cell-derived ONNV displays a high PE content

To explore whether ONNV contains phospholipids that mediate TIM-1 binding, we performed lipidomics analysis of purified BHK-21- and C6/36-derived ONNV. Since PC, PE, PS, and cholesterol (Chol) are the main phospholipid components of cellular membranes, we focused on these lipid classes. PS was detected in both BHK-21- and C6/36-derived ONNV, accounting for 6.71 and 14.26 mol% of total lipids, respectively **(**Figure 4(A), S4**)**. C6/36-derived ONNV particles had a significantly higher PE content (50.92 mol% of total lipids) and lower Chol content (19.86 mol% of total lipids) as compared to BHK-21-derived ONNV (1.67 mol% PE, 49.58 mol% Chol) **(**Figure 4(A), S4). In short, mosquito cell-derived ONNV contained a higher percentage of PE and PS than mammalian cell-derived virions.

To confirm that PE is present on the outer leaflet of the viral envelope of C6/36-derived ONNV, we incubated virus stocks with duramycin-LC-biotin, a PE-binder previously utilized to prove interaction between TIM-1 and flavivirus-PE [20]. Duramycin binding reduced infection with C6/36-derived ONNV on HEK TIM-1, but not with BHK-21-derived ONNV nor non-enveloped AdV5 (Figure S5(A)). ONNV infection was also reduced on TIM-1-negative HEK Mxra8 cells (Figure S5(B)), which disqualifies duramycin as a tool to prove TIM-1-ONNV/PE interactions. However, the data support that duramycin binds to C6/36-derived ONNV and thus provide evidence of PE presence on the outer leaflet of C6/36-derived ONNV.

### PS and PE can both engage TIM-1 during ONNV entry

Next, we used a liposome competition assay to examine the role of PE and PS in mosquito cell-derived virion infectivity. Virion-associated PE can, similar to PS, serve as a MILIBS ligand for human TIM-1 [38]. We generated liposomes, in which we replaced PS or PE or both with PC (Figure 4(B)). Prior to infection, we incubated HEK WT and HEK TIM-1 cells with increasing concentrations (1 μM to 100 μM) of liposomes. Apart from PC:Chol liposomes, which lack PS and PE, all liposome formulations competed with C6/36-derived ONNV early during HEK TIM-1 infection (Figure 4(C)). PC:PE:Chol liposomes, devoid of PS, blocked ONNV infection similarly to liposomes containing PS (Figure 4(C)). As expected, liposomes competed neither with C6/36-derived nor with BHK-21-derived ONNV during infection of HEK WT (Figure S6 (A and C)). Surprisingly, inhibition of BHK-21-derived ONNV infection by liposomes was less pronounced, with only competition with PC:PE:Chol liposomes significantly reducing infection levels (Figure S6(B)).

To determine whether PE can act as the sole mediator of ONNV entry in absence of PS, we incubated virions with increasing concentrations of PS decarboxylase, which converts PS to PE [19]. Both BHK-21-derived ONNV and C6/36-derived ONNV were rendered less infectious upon PS depletion (Figure 4(D)), indicating that viral envelope PS is necessary for virion-TIM-1 interactions during the entry/fusion process. Overall, these data suggest that, while TIM-1 can engage both PS and PE, viral envelope PS remains important during TIM-1-mediated entry of mosquito cell-derived ONNV.

### ONNV infection is TIM-1 dependent in cells naturally expressing TIM-1

**Figure 5.**
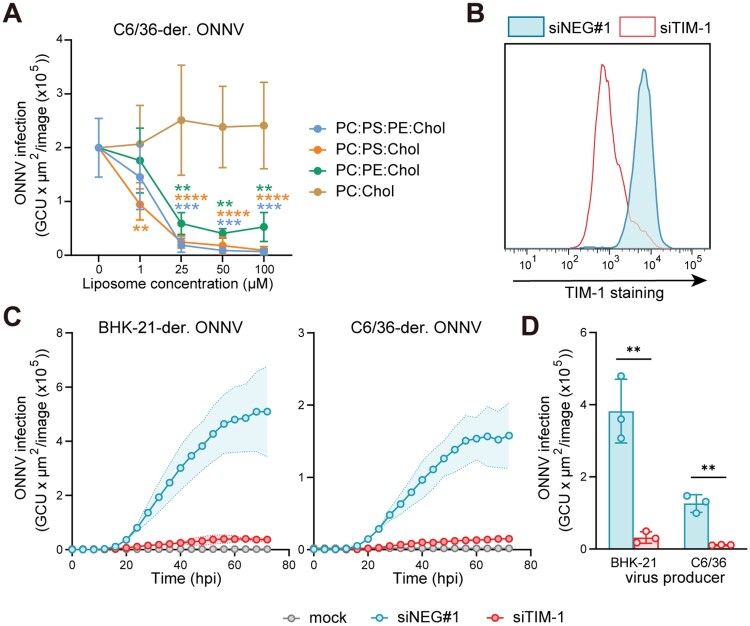
ONNV infection is TIM-1-dependent in cells naturally expressing TIM-1. (A) Liposome inhibition of ONNV entry into A549ΔMAVS cells (MOI:4), assessed by live cell imaging. Inoculum containing ONNV and liposomes was removed at 4 hpi. Data show mean infection levels ± SD at 48 hpi of three biological replicates. One-way ANOVA with Dunnett’s compared with untreated control. (B) Confirmation of TIM-1 knockdown in A549ΔMAVS cells treated with non-targeting siRNA (siNEG#1) or an siRNA pool targeting TIM-1 (siTIM-1), analysed by flow cytometry at 48 h. Histogram is representative of three biological replicates. (C) Time-course of ONNV infection in A549ΔMAVS (MOI:4) transfected with siNEG#1 or siTIM-1, assessed by live cell imaging. Data show mean ± SD of three biological replicates. (D) ONNV infection levels at 48 hpi from (C). Unpaired, two-tailed *t*-test.

Next, we validated the importance of TIM-1 in ONNV infection of cells endogenously expressing TIM-1. We repeated the liposome competition assay on TIM-1 positive A549ΔMAVS, which are susceptible to ONNV whereas WT A549 are refractory. All liposome formulations containing PS and/or PE inhibited ONNV infection **(**Figure 5(A), Figure S6(D)**)**, indicating the presence of a PS receptor that engages with ONNV envelope PS or PE during entry. To confirm that TIM-1 is the PS receptor mediating ONNV uptake, we performed siRNA-mediated knockdown of TIM-1 **(**Figure 5(B)). TIM-1 knockdown was detrimental to both mammalian cell- and mosquito cell-derived ONNV infection in these cells, confirming the importance of TIM-1 in a physiological context (Figure 5(C and D)).

## Discussion

In this study we investigated the role of TIM-1 as an attachment factor at the mosquito-mammalian interface of alphavirus transmission. Upon assessment of virus infectivity in HEK cells with and without TIM-1 expression, we found that most mosquito cell-derived alphaviruses showed similar TIM-1 dependency as mammalian cell-derived viruses, including for the first time, ONNV. We observed that ONNV primarily relies on TIM-1 in the absence of a primary alphavirus receptor. The phospholipid binding pocket was critical for TIM-1 dependency of both mammalian and mosquito cell-derived ONNV. Collectively, our data indicate that TIM-1 ligands are displayed on virions produced in mosquito cells. Mass spectrometry-based lipidomics revealed the presence of PS, as well as a high PE content, in mosquito cell-derived ONNV. Finally, liposome competition and PSD treatment indicated that both PE and PS serve as TIM-1 ligand but emphasized the important role of PS on the viral envelope.

HEK cells do not express Mxra8, but do express very low-density lipoprotein receptor (VLDLR) [7] and low-density lipoprotein receptor class A domain-containing 3 (LDLRAD3) [8], which are receptors for SFV and VEEV, respectively. Correspondingly, we observed no TIM-1 dependency for SFV and VEEV. This suggests that the TIM-1 entry route is relevant in cells which lack primary receptors. We experimentally tested this theory by demonstrating that TIM-1 expression does not further enhance ONNV infection in Mxra8-overexpressing HEK cells. This corresponds to a similar observation for SARS-CoV-2, i.e. that the impact of TIM-1-mediated uptake is greater in cells that harbour low ACE2 surface levels [39].

Richard et al. and others demonstrated that human TIM-1 binds to both PS and PE liposomes [20,38]. Using duramycin, they demonstrated that PE exposed on virions mediates TIM-1-dependent enhancement of infection with Ebola, dengue, and West Nile viruses [20,40]. Surprisingly, when we repeated this experiment with ONNV, we found that this duramycin-blocking effect was independent of TIM-1 and was not due to disruption of the viral lipid envelope (Figure S5(C)). This suggests that duramycin-LC-biotin sterically interferes with alphavirus-receptor interactions or otherwise reduces viral fusion. This likely reflects structural differences between alphaviruses, flaviviruses, and filoviruses. Consequently, we could not use duramycin as evidence of direct-virion-PE-TIM-1 binding, but only prove exposure of PE on ONNV particles. In line with previous observations that TIM-1 can bind PE, PC:PE:Chol liposomes competed with ONNV on HEK TIM-1 and endogenous TIM-1 expressing cells. This confirms that human TIM-1, in addition to showcasing PS affinity, also binds PE.

The high levels of outer leaflet PE in mosquito-derived ONNV reflect the high PE-content of insect cells [25]. Yet, PS decarboxylase treatment of ONNV virions reduced infection in HEK TIM-1 cells, which matches data from Groβ et al. who had treated Zika virus with PSD and saw a similar reduction in infection [19]. This suggests that PS in the viral envelope is an important TIM-1 ligand during ONNV infection. As an additional layer of complexity, PE and PS synergize. Presence of PE on the same membrane as PS results in stronger PS binding to PS receptors than in the absence of PE [41]. Consequently, high PE content of mosquito cell-derived ONNV may support PS-TIM-1 interactions. Further studies investigating PE/PS synergy of mosquito-derived alphaviruses and determining the ratios of those lipids ideal for infection could elucidate how viral envelope PE could facilitate arbovirus infection at the mosquito-mammalian interface.

One limitation of this study is that C6/36 cells are derived from *Aedes albopictus*, which is not the main vector species of ONNV, i.e. *Anopheles gambiae*. Here we used C6/36 because they lack Dicer2 activity [42], which disrupts the main antiviral response of insect cells [43]. Hence, C6/36 cells are highly permissive and routinely used in arbovirus studies. In line with this, we observed enhanced alphavirus propagation in C6/36 compared to Aag2-AF5, which can likely be attributed to the presence of a strong RNA interference response in the latter. Studies with virions derived from vector-matched mosquito saliva or mosquito midgut [44] are warranted to verify lipid envelope composition and TIM-1 dependency at the mosquito-mammalian interface. Additionally, this study employed HEK TIM-1 and A549ΔMAVS as TIM-1-positive recipient cells, whereas a human skin model would be more representative of mosquito to human transmission. In human skin, TIM-1 expression is specific to the basal layer of the epidermis, which is challenging to recapitulate in a cell culture model [9,45,46]. Future studies on advanced human skin models that reflect TIM-1 positivity of the basal epidermis will clarify the contribution of TIM-1 and other PS receptors in virus transmission.

This study attempted to better understand the role of human lipid receptors and alphavirus envelope composition at the mosquito-mammalian interface *in vitro*. Several studies have described TIM-1’s role in enhancing entry of enveloped viruses ranging from flaviviruses and coronaviruses to filoviruses and arenaviruses [9,17-20,39,46-48]. Whether other mosquito-derived viruses also interact with TIM-1 through mosquito cell membrane-derived PE and PS will be part of future investigations. Especially in light of recent reports on extracellular vesicles carrying replication-competent flaviviral nucleocapsids in mosquitoes [49], TIM-1 may play a major role. Furthermore, this study investigated ONNV, a poorly characterized alphavirus that is closely related to CHIKV and that is likely underdiagnosed due to suboptimal differential diagnostics. Similar to CHIKV, ONNV engages the receptor Mxra8 [6], but no other entry factor was previously reported. Here, we demonstrate that TIM-1 enhances ONNV infection by engaging phospholipids exposed on virions of both vertebrate and invertebrate origin.

## Supplementary Material

Supplemental Material

Supplemental Material

Supplemental Material

Supplemental Material

Supplemental Material

Supplemental Material

Supplemental Material
