# Supplementary material for: TIM-1 promotes infection with mosquito cell-derived alphaviruses through virion-associated phospholipids
Source: Emerg Microbes Infect. 2026 May 13;15(1):2673648. doi: 10.1080/22221751.2026.2673648 (PMC13250875; doi:10.1080/22221751.2026.2673648)
Supplement: Supplemental Material [file TEMI_A_2673648_SM5754.docx]

**TIM-1 promotes infection with mosquito cell-derived alphaviruses through virion-associated phospholipids**

Ju Eun Yoo^1,2, †^, Anja C. M. de Bruin^6, †^, Hanna Reßin^3^, Mindaugas Paužuolis^6^, Lifeng Liu^6^, Anja Moosmann^3^, Miriam Becker^1,2^, Lisa Lasswitz^1,2^, Rüdiger Groß^3^, Gisa Gerold^1,2,4,5,6,*^

^1^Institute of Biochemistry, University of Veterinary Medicine Hannover, Hannover, Germany; ^2^Research Centre for Emerging Infections and Zoonoses, University of Veterinary Medicine Hannover, Hannover, Germany; ^3^Institute of Molecular Virology, Ulm University Medical Center, Ulm, Germany; ^4^Department of Clinical Microbiology, Umeå University, Umeå, Sweden; ^5^Wallenberg Centre for Molecular Medicine (WCMM), Umeå University, Umeå, Sweden; ^6^Institute of Virology, Medical University of Innsbruck, Innsbruck, Austria; ^*^Corresponding author

† These authors contributed equally to this work

*Corresponding author: gisa.gerold@i-med.ac.at

Prof. Dr. Gisa Gerold

Schöpfstraße 41

6020 Innsbruck, Austria

+43 512 9003 71701

TIM-1 promotes infection with mosquito cell-derived alphaviruses through virion-associated phospholipids

# Supplementary methods

## Lipidomics

### ONNV sample preparation

P1 ONNV was harvested from BHK-21 and C6/36 cells infected at MOI:0.1, as described in ***Virus production and titration*** subsection of the main **Materials and methods**. Pooled supernatants were overlaid on 20% w/v sucrose dissolved in HNE buffer (10 mM HEPES, 150 mM NaCl, 1 mM EDTA, pH 7.4) and centrifuged at 75,000 × g, 4 h at 4 °C (Avanti J-26S XP, Beckman Coulter). Pellets were resuspended in HNE buffer and inactivated using a UV crosslinker for 5 min at 0.999 kJ/cm^2^ (Analytik Jena) on a cold block.

### Lipid extraction for mass spectrometry lipidomics

Mass spectrometry-based lipid analysis was performed by Lipotype GmbH (Dresden, Germany) as described by Surma et al. 2021 [1]. Lipids were extracted using a chloroform/methanol procedure [2]. After extraction, the organic phase was transferred to an infusion plate and dried in a speed vacuum concentrator. The dry extract was re-suspended in 7.5 mM ammonium formate in chloroform/methanol/propanol (1:2:4, V:V:V).

### Mass spectrometry lipidomics

Samples were analysed by Lipotype GmbH (Dresden, Germany) by direct infusion on a QExactive mass spectrometer (Thermo Scientific) equipped with a TriVersa NanoMate ion source (Advion Biosciences). Samples were analysed in both positive and negative ion modes with a resolution of Rm/z=200=280000 for MS and Rm/z=200=17500 for MSMS experiments, in a single acquisition. MSMS was triggered by an inclusion list encompassing corresponding MS mass ranges scanned in 1 Da increments [3]. Both MS and MSMS data were combined to monitor CE, Chol, DAG and TAG ions as ammonium adducts; LPC, LPC O-, PC, PC O-, as formate adducts; and CL, LPS, PA, PE, PE O-, PG, PI and PS as deprotonated anions. MS only was used to monitor LPA, LPE, LPE O-, LPG and LPI as deprotonated anions; Cer, HexCer and SM as formate adducts and cholesterol as ammonium adduct of an acetylated derivative.

### Lipidome analysis

Data were analysed by Lipotype GmbH (Dresden, Germany) with an in-house developed lipid identification software based on LipidXplorer [4,5]. Post-processing and normalization of data were performed using an in-house developed data management system. Only lipid identifications with a signal-to-noise ratio >5, and a signal intensity 5-fold higher than in corresponding blank samples were considered for further data analysis. Lipid class percentages and total lipid amount were visualised using RStudio (Build 748) and R (version 4.5.0). Principal component analysis (PCA) was carried out on lipid class mole percentages using the prcomp( ) function and the results were visualised using the autoplot function. Differential expression analysis of lipid class mole percentages was performed using student's T-test in Perseus (v1.6.15.0). Bar plots of total lipid amounts detected in the samples were visualised using the tidyverse package.

# Supplementary figures

S1


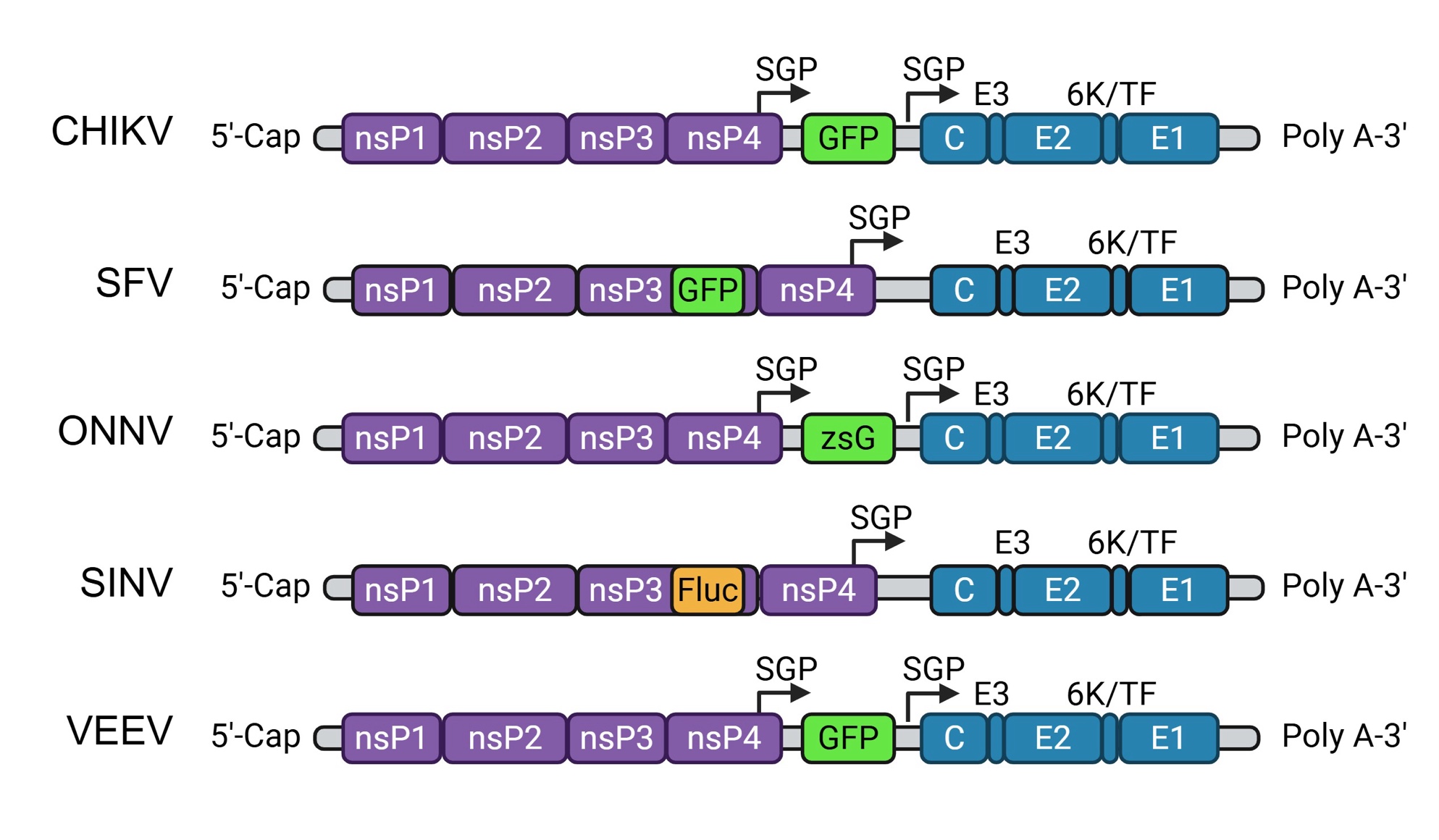


S2


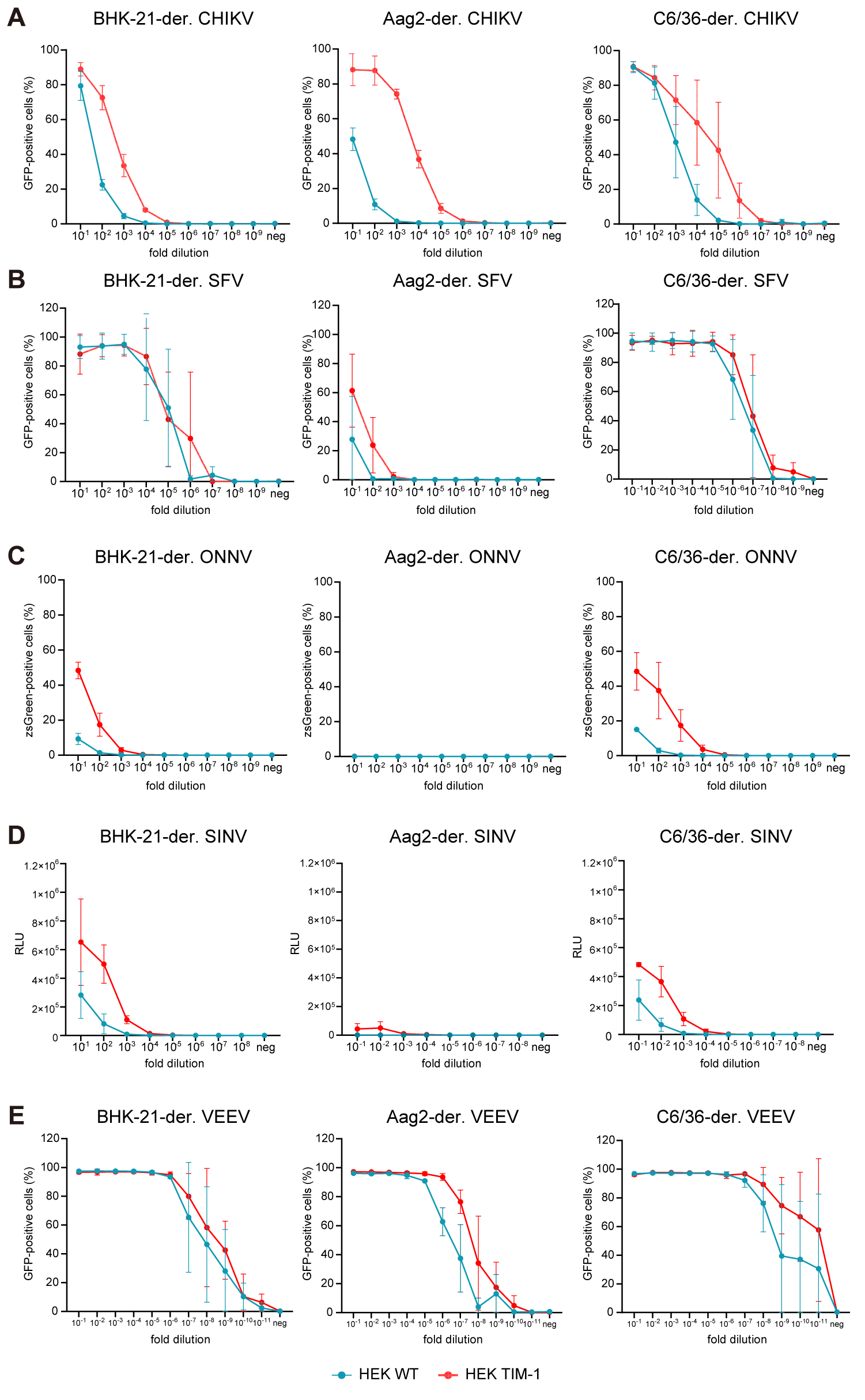


S3


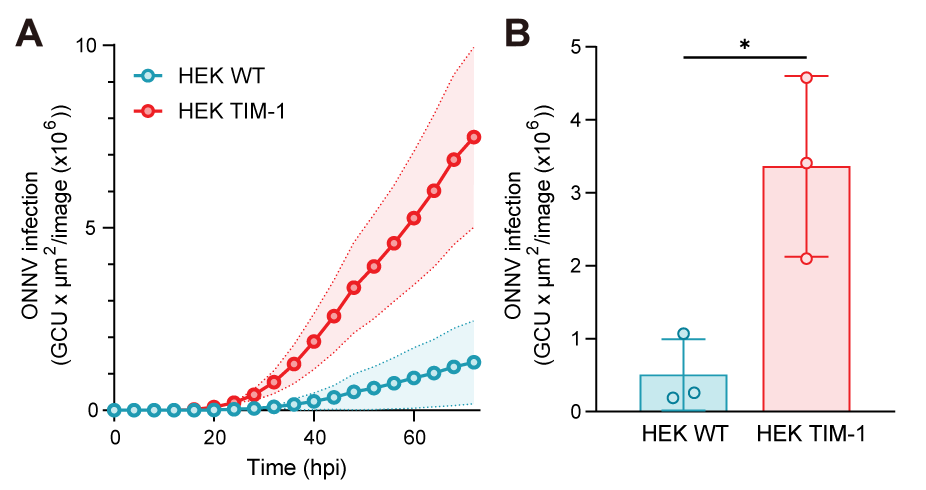


S4


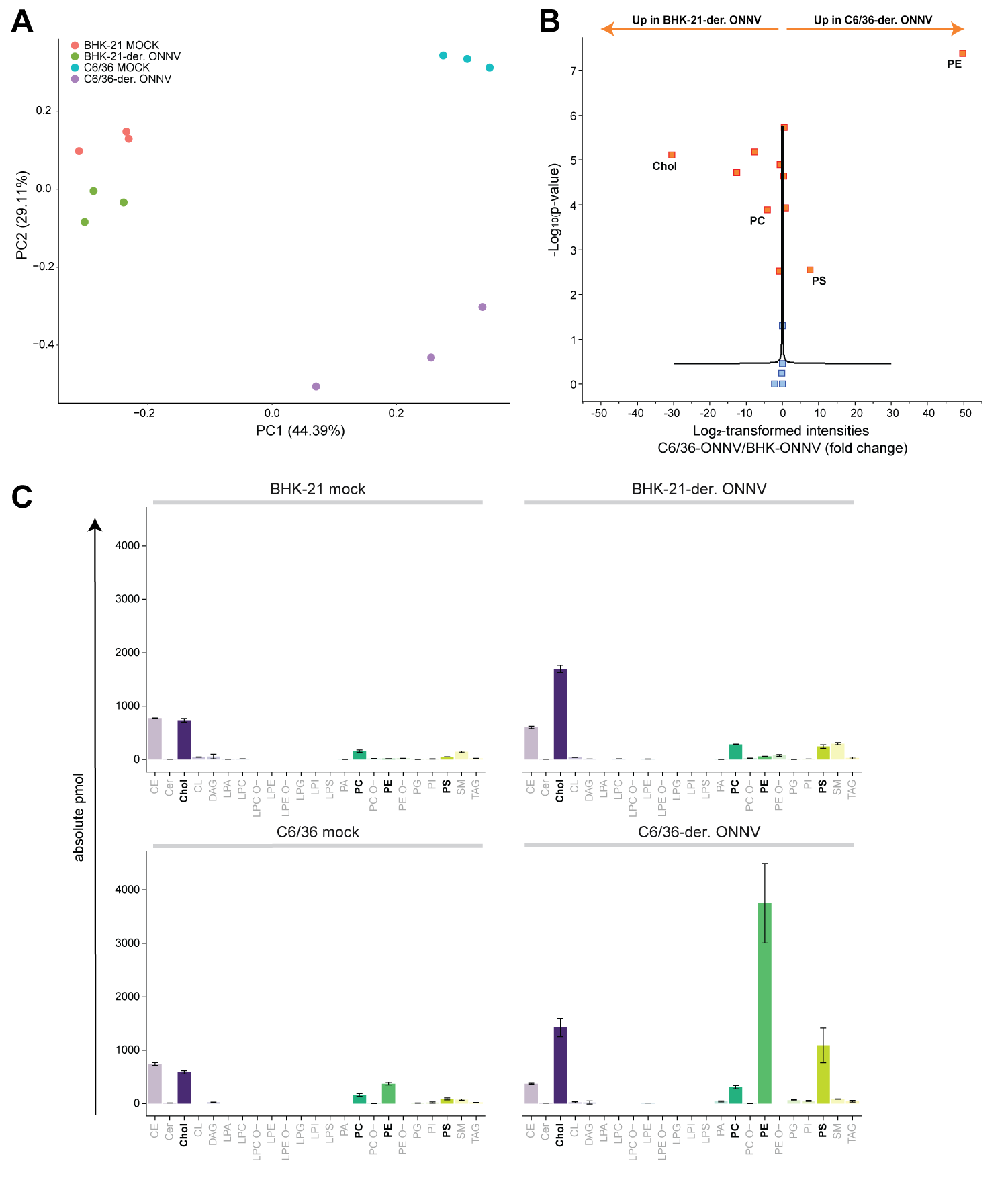


S5


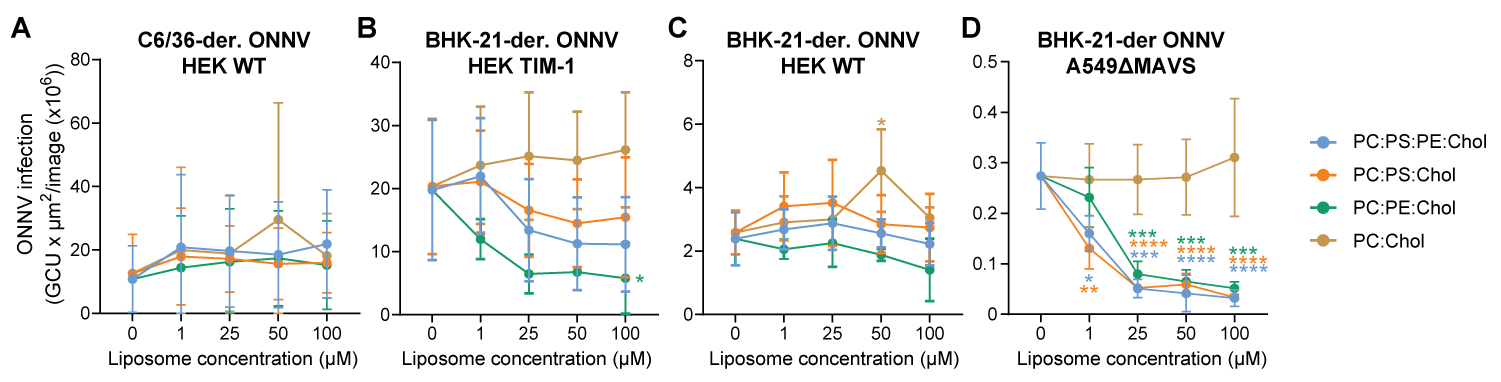


S6


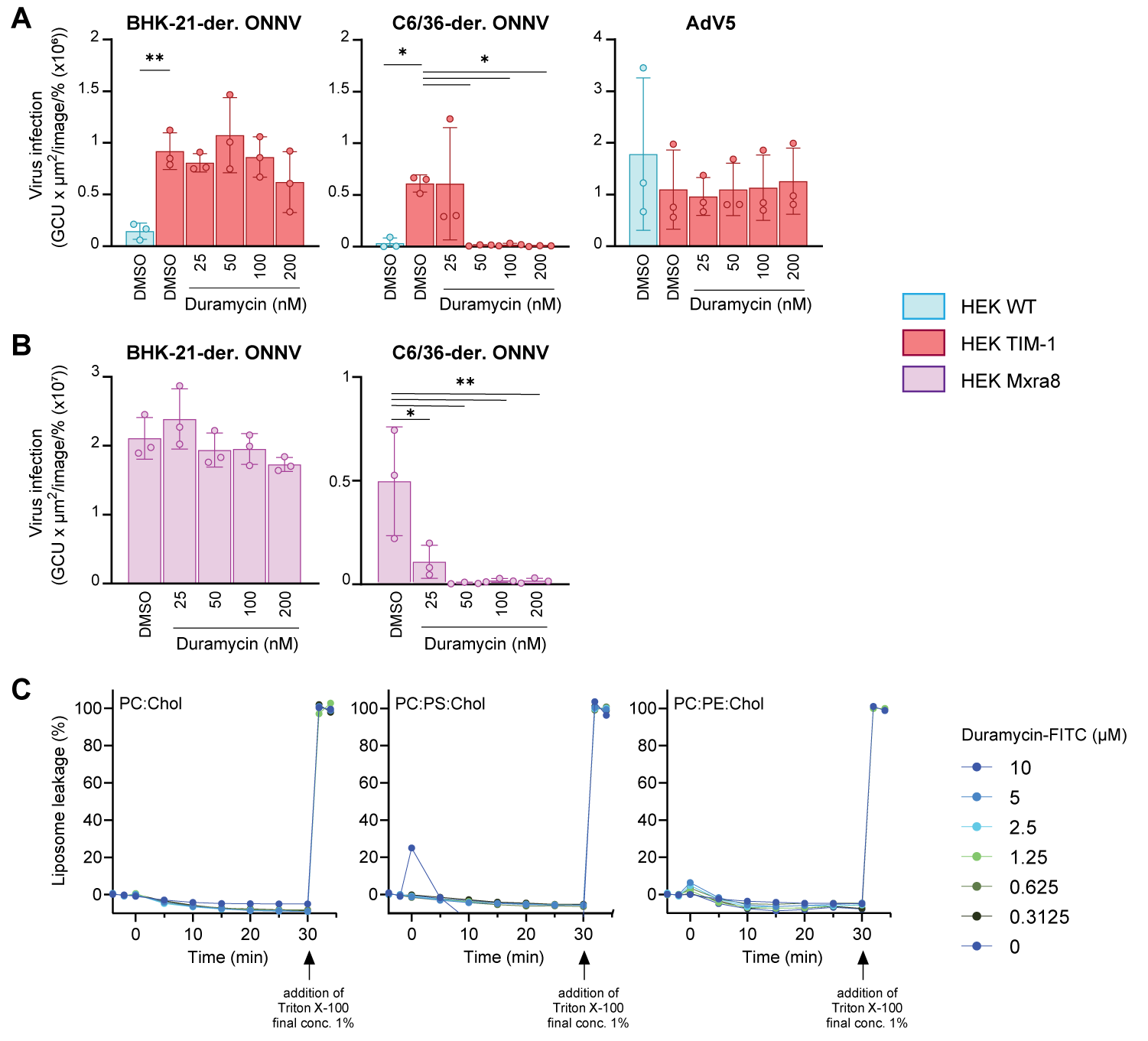


# Figure captions

Figure S1. Schematic representation of full-length alphavirus infectious clones used in the study. CHIKV (LR2006 OPY1), ONNV (Chad), and VEEV (TC-83) clones express GFP or zsGreen under a subgenomic promoter. SFV (SFV4) and SINV (Toto1101) clones encode GFP or firefly luciferase in frame with nsP3, inserted at its C-terminus.

Figure S2. Titration curves of (A) CHIKV, (B) SFV, (C) ONNV, (D) SINV, and (E) VEEV derived from the indicated mammalian (BHK-21) or mosquito cell line (Aag2-AF5 or C6/36) on HEK WT and HEK TIM-1. Data are mean ± SD of three biological replicates.

Figure S3. TIM-1 expression enhances infection of human cell-derived ONNV. (A) Time-course of human dermal fibroblast-derived ONNV infection of HEK WT and HEK TIM-1, assessed by live cell imaging. Data are mean ± SD of three biological replicates. (B) ONNV infection levels at 48 hpi from the experiment in (A). Unpaired, two-tailed t-test.

Figure S4. Details of the lipidomics analysis. (A) Principal Component Analysis (PCA) of BHK-21-derived and C6/36-derived ONNV and their respective mock-infected controls. Lipid class mol percentage fractions of the total sample were used as input data. (B) Volcano plot of differential abundance of lipid classes in BHK-21-derived and C6/36-derived ONNV. Lipid class mol percentage fractions of the total sample were used as input data. Data were analysed using a student’s t-test with an FDR of 0.05. Differentially abundant lipid classes of interest are labelled. Differentially abundant lipid classes are shown in red and non-differentially abundant lipid classes are shown in blue. (C) Total measured lipid class amounts from non-normalized samples. Data represent the mean ± SD from three technical replicates. Lipid classes of interest are highlighted.

Figure S5. Liposome-mediated inhibition of C6/36-derived (C6/36-der.) and BHK-21 derived (BHK-21-der.) ONNV infection of HEK WT, HEK TIM-1, and A549ΔMAVS. (A) HEK WT were inoculated with C6/36-derived ONNV at MOI:0.5 in the presence of the indicated liposome formulations and concentrations. Infection levels were assessed by live cell imaging (24 hpi data are shown). HEK TIM-1 (B) or HEK WT (C) were inoculated with BHK-derived ONNV at MOI:1 in the presence of liposomes and infection levels were assessed as in (A). (D) A549ΔMAVS were inoculated with BHK-derived ONNV at MOI:4 in the presence of liposomes. Inoculum was removed at 4 hpi, and infection levels were assessed by live cell imaging (48 hpi data are shown). Data are mean ± SD from three biological experiments. One-way ANOVA with Dunnett’s compared with untreated control.

Figure S6. Duramycin blocks entry of C6/36-derived ONNV into HEK cells via TIM-1 independent mechanism. (A, B) ONNV and AdV5 stocks were incubated with increasing amounts of Duramycin-LC-Biotin and subsequently used to inoculate HEK WT or HEK TIM-1 (A) or HEK Mxra8 (B). Virus infection levels were assessed by live cell imaging (24 hpi data are shown). Data are mean ± SD from three biological experiments. One-way ANOVA with Dunnett’s compared with vehicle control. (C) Increasing concentrations (0-10 mM final concentration) of duramycin were added to Sulforhodamine B-containing PC:Chol, PC:PS:Chol, PC:PE:Chol liposomes. Liposome leakage was measured by Sulforhodamine B fluorescence in the PBS. Baseline fluorescence was measured on wells with liposomes alone, and 100% leakage was measured by addition of TritonX-100 (1% final concentration).

# References

[1] Surma MA, Gerl MJ, Herzog R, et al. Mouse lipidomics reveals inherent flexibility of a mammalian lipidome. Sci Rep. 2021;11(1):19364.

[2] Ejsing CS SJ, Surendranath V, Duchoslav E, Ekroos K, Klemm RW, Simons K, Shevchenko,A. Global analysis of the yeast lipidome by quantitative shotgun mass spectrometry. Proc Natl Acad Sci USA. 2009;106(7):2136-2141.

[3] Surma MA, Herzog R, Vasilj A, et al. An automated shotgun lipidomics platform for high throughput, comprehensive, and quantitative analysis of blood plasma intact lipids. Eur J Lipid Sci Technol. 2015;117(10):1540-1549.

[4] Herzog R, Schuhmann K, Schwudke D, et al. LipidXplorer: a software for consensual cross-platform lipidomics. PLoS One. 2012;7(1):e29851.

[5] Herzog R, Schwudke D, Shevchenko A. LipidXplorer: Software for Quantitative Shotgun Lipidomics Compatible with Multiple Mass Spectrometry Platforms. Curr Protoc Bioinformatics. 2013;43:14.12.1-14.12.30.
