# Supplementary figures and images for: TIM-1 promotes infection with mosquito cell-derived alphaviruses through virion-associated phospholipids
Source: Emerg Microbes Infect. 2026 May 13;15(1):2673648. doi: 10.1080/22221751.2026.2673648 (PMC13250875; doi:10.1080/22221751.2026.2673648)

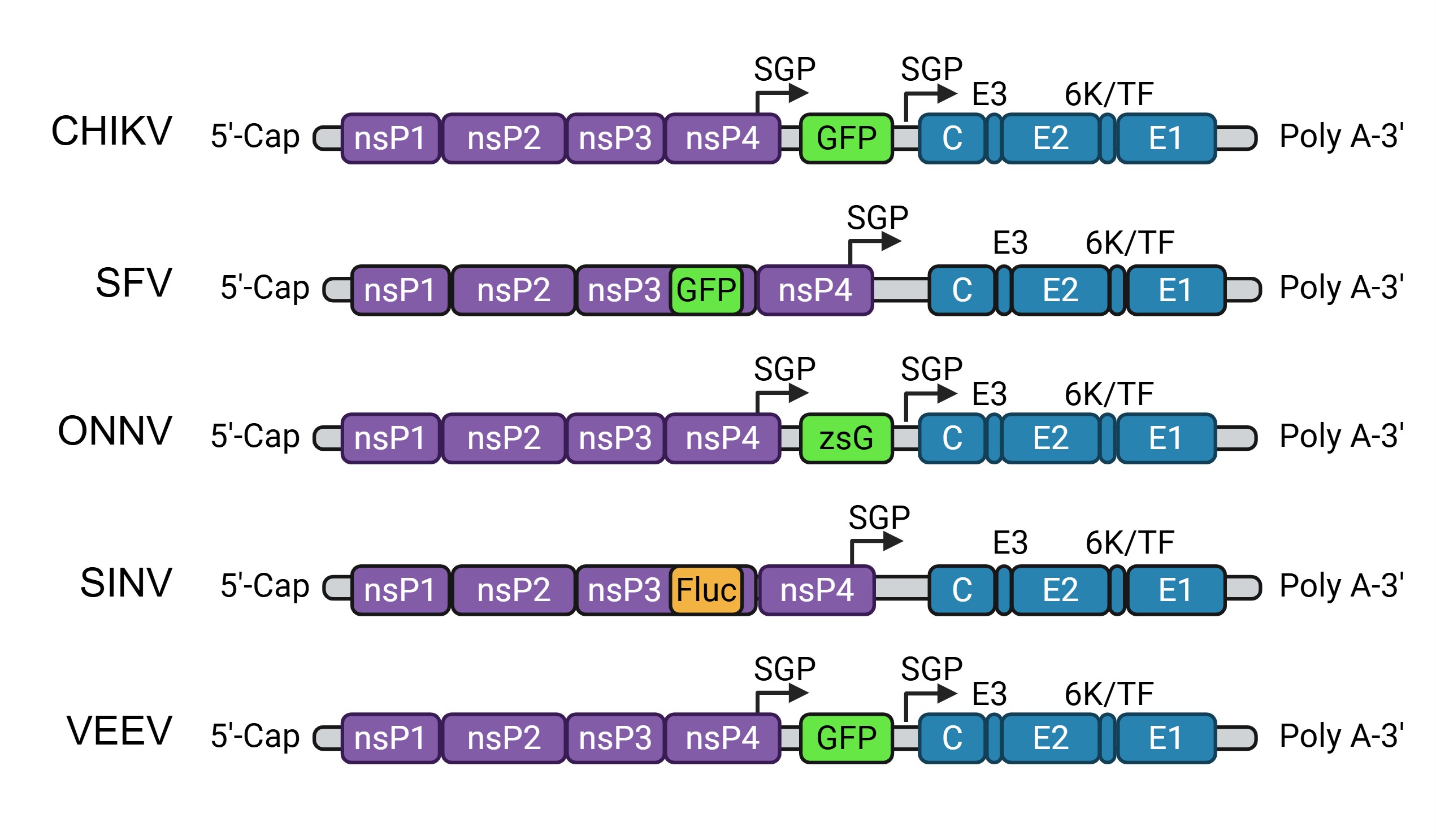

Supplement: Supplemental Material [file TEMI_A_2673648_SM4475.jpeg]

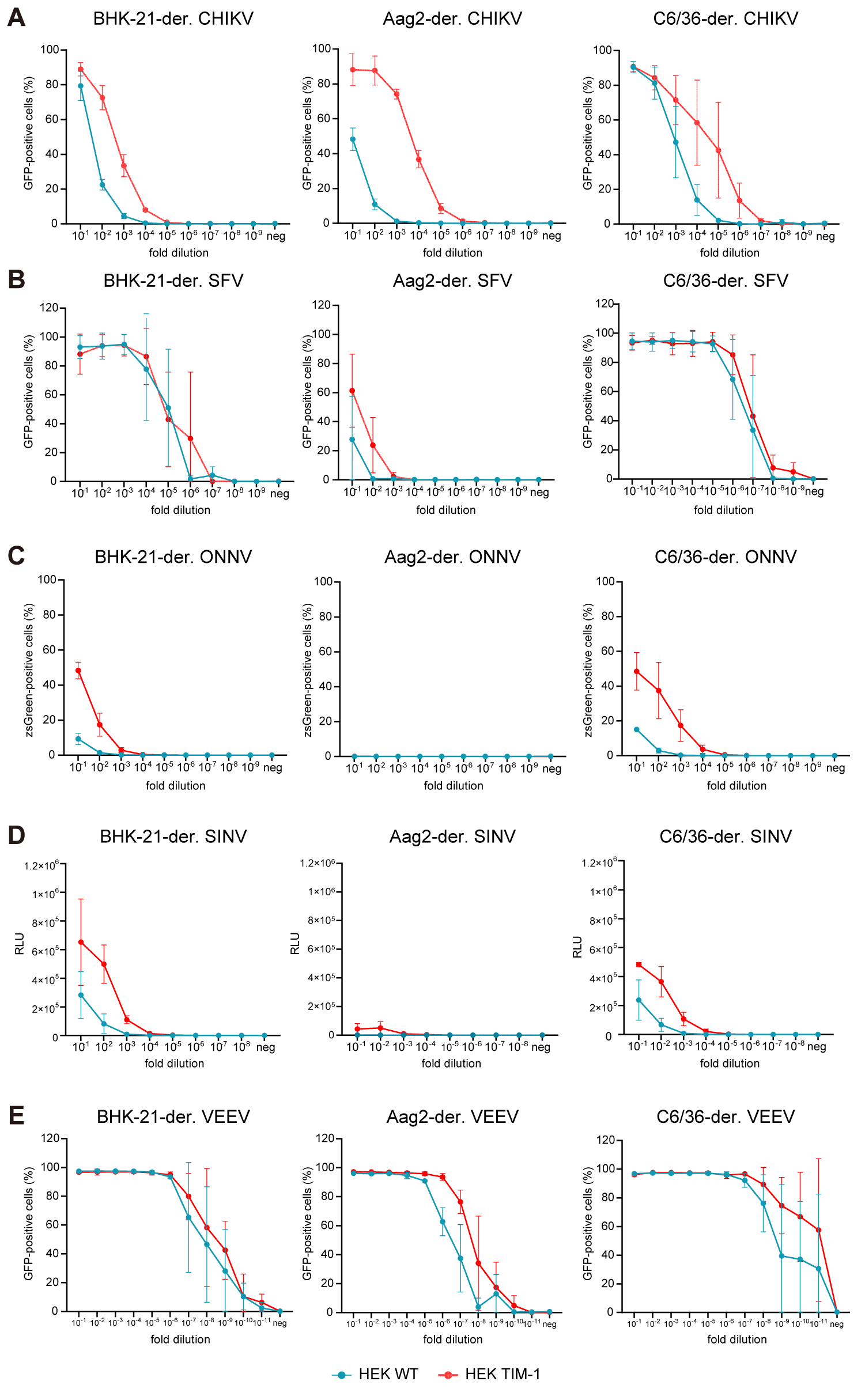

Supplement: Supplemental Material [file TEMI_A_2673648_SM4460.tif]

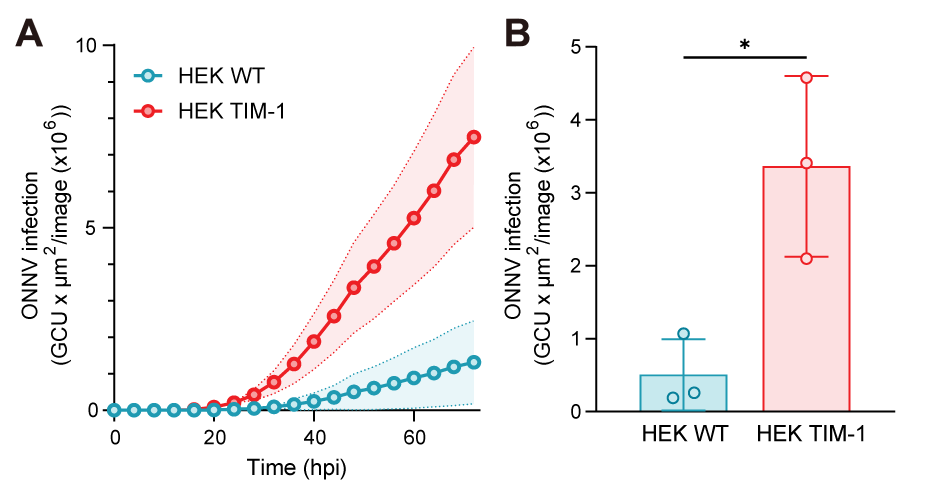

Supplement: Supplemental Material [file TEMI_A_2673648_SM4410.tif]

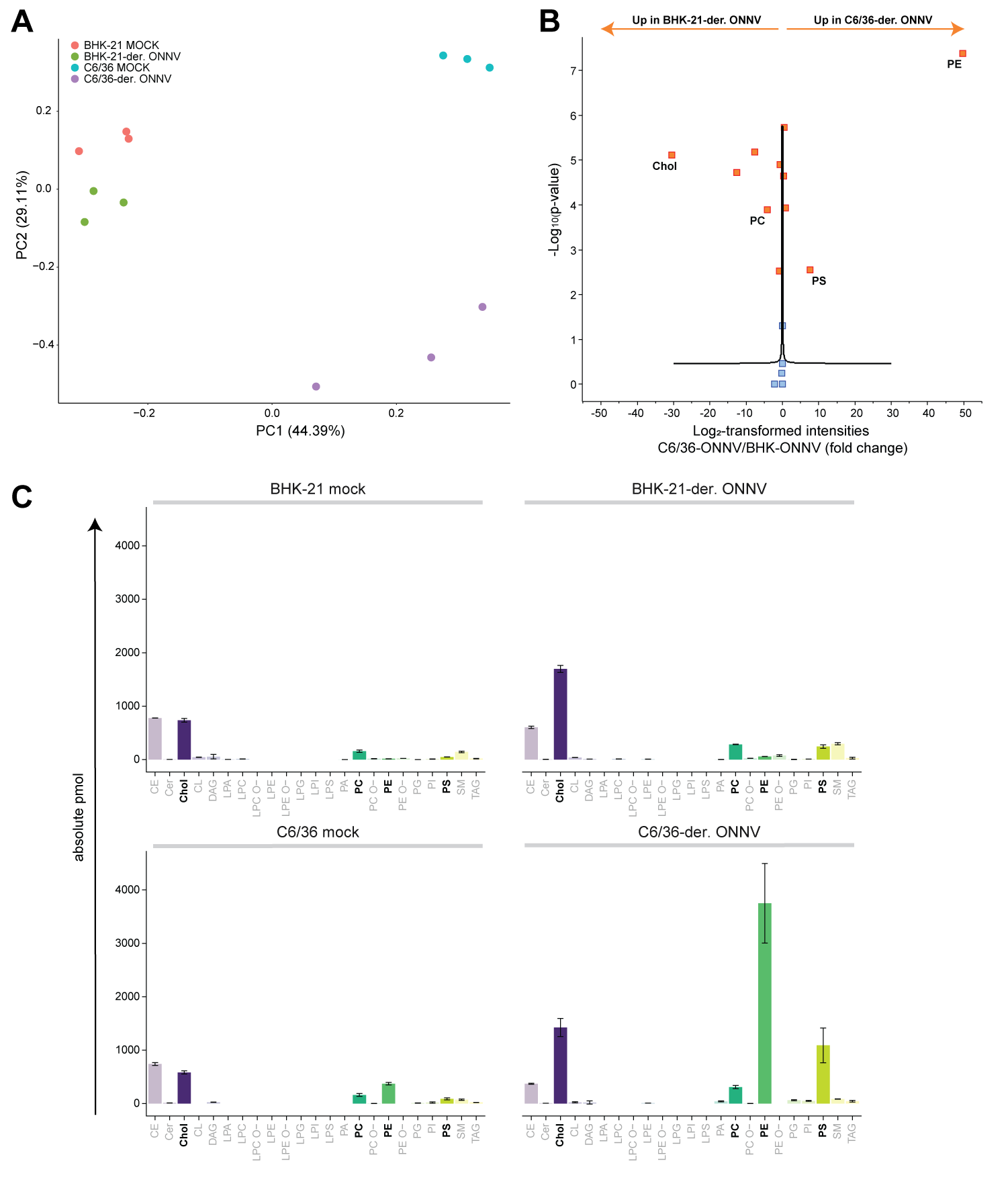

Supplement: Supplemental Material [file TEMI_A_2673648_SM4397.tif]

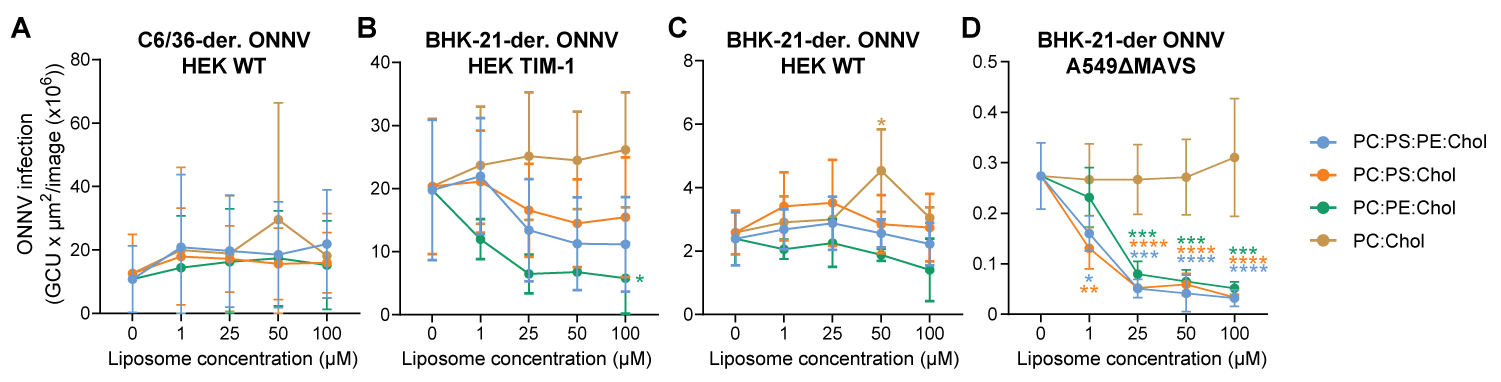

Supplement: Supplemental Material [file TEMI_A_2673648_SM4384.tif]

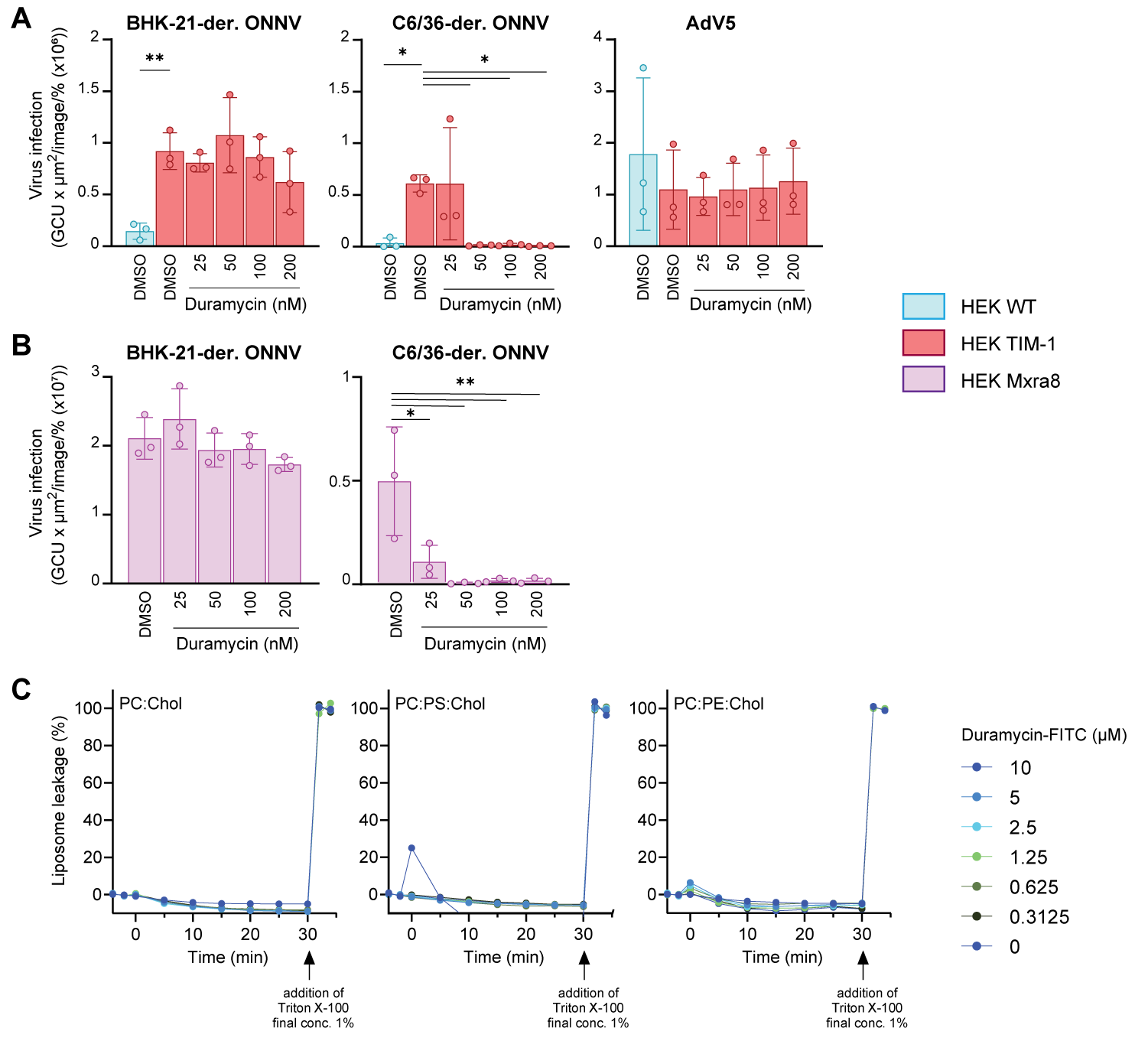

Supplement: Supplemental Material [file TEMI_A_2673648_SM4371.tif]
